# A survey of travel behaviour among scientists in Germany and the potential for change

**DOI:** 10.7554/eLife.56765

**Published:** 2020-05-28

**Authors:** Verena Haage

**Affiliations:** Max Delbrück Center for Molecular Medicine, Helmholtz AssociationBerlinGermany

**Keywords:** research culture, conferences, travel, future conference formats, sutainability, climate change, Human

## Abstract

Awareness of the environmental impact of conferences is growing within the scientific community. Here we report the results of a survey in which scientists in Germany were asked about their attendance at conferences, their reasons for attending, and their willingness to explore new approaches that would reduce the impact of conferences on the environment. A majority of respondents were keen to reduce their own carbon footprint and were willing to explore alternatives to the traditional conference.

## Introduction

Scientists attend conferences to present their results, to hear results from other scientists, and to meet other people in their field. Going to a conference often involves long-distance air travel, so the benefits of attending need to be weighed up against the environmental cost of attending (Rosen, 2017). In March 2019, inspired by the student-run environmental movement Fridays for Future, a group of scientists from Germany, Austria and Switzerland founded Scientists for Future to encourage the scientific community to take action against climate change.

As scientists, we have a responsibility to drive evidence-based societal and cultural change. Various ideas for reducing the carbon footprint of research have been proposed, such as exploring alternatives to flying (Nature Nanotechnology, 2019) and making scientific conferences more sustainable (Hamant et al., 2019). Here we report the results of a survey that was conducted to gain some insight into the attitude of scientists to conferences and travel, and to gauge their appetite for change. Since the survey was completed, the COVID-19 pandemic has resulted in the cancellation of many conferences and a significant reduction in air travel around the world. It seems unlikely that scientists will travel as much in the future as they did before the COVID-19 pandemic, so the results of this survey are a sort of snapshot of the attitude of scientists in a European country to conferences and travel before the pandemic.

## Results

### Conference attendance increases with career stage

**Figure 1.**
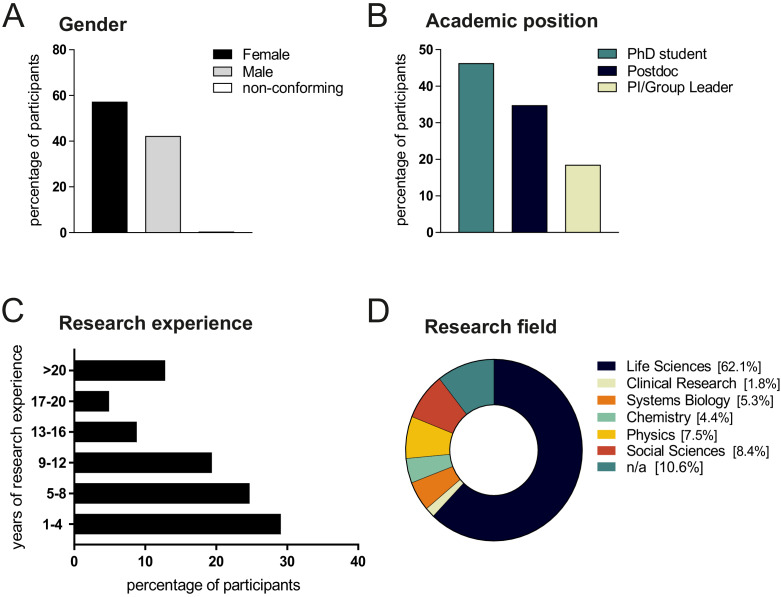
Survey demographics. Distribution of respondents by gender (**A**), career stage (**B**), years of research experience (**C**), and research field (**D**). Seven of the research areas asked about in the survey – Life Science, Neuroscience, Immunology, Microbiology, Genetics, Cancer Biology and Cardiovascular/Metabolic research – were combined into a single Life Sciences research area during analysis. Figure 1—source data 1.Survey participants’ genders, career stages, years of research experience and research fields.

Data were collected from 227 scientists currently living and working in Germany: the original aim was to study an international sample, but more than 80% of the responses came from scientists based in Germany (see Methods for information on how the survey was disseminated). There were more responses from women than from men (57% vs 42%; see Figure 1A). The breakdown according to career stage was: doctoral researchers/PhD students (46%); postdoctoral researchers (35%); independent group leaders/principal investigators (PIs; 19%; see Figure 1B,C). The breakdown according to scientific discipline was: Life Sciences (62%); Social Sciences (8%); Physics (8%); Systems Biology (5%); Chemistry (4%); Clinical Research (2%; see Figure 1D). The remaining 11% of participants did not answer this question.

**Figure 2.**
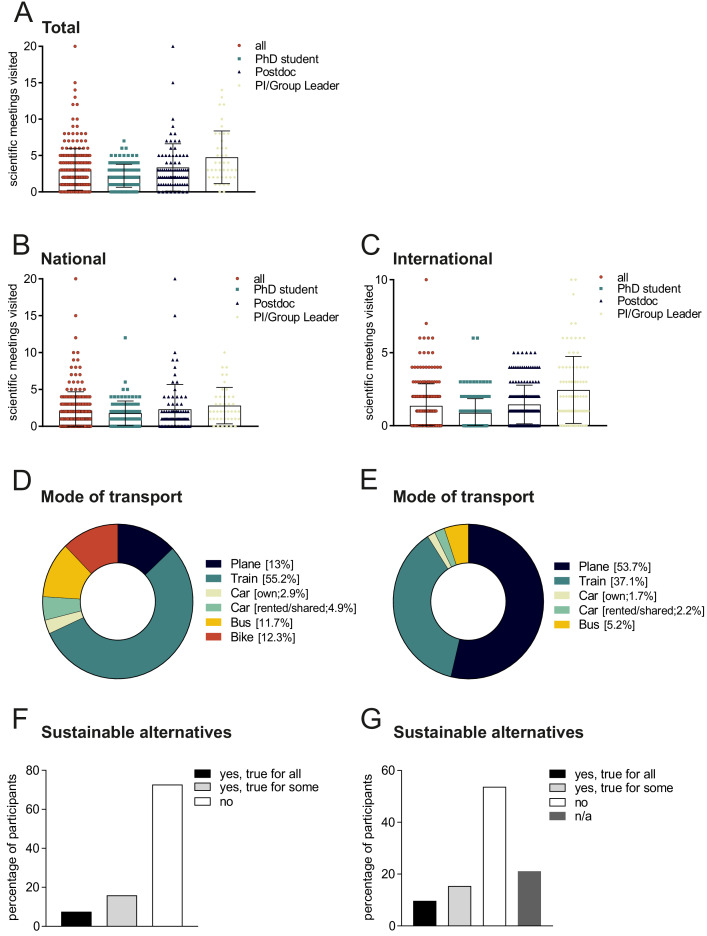
Travelling to national and international conferences. (**A**) Total number of conferences attended in 2019 by all respondents (red), by doctoral researchers/PhD students (cyan), by postdoctoral researchers (black), and by PIs/group leaders (chartreuse). Each dot represents one respondent; mean ± SD. Number of national conferences (**B**) and international conferences (**C**) attended in 2019 by all scientists (red), by doctoral researchers/PhD students (cyan), by postdoctoral researchers (black), and by PIs/group leaders (chartreuse). Mode of transport used for travelling to national conferences (**D**) and international conferences (**E**). Answers to the question ‘could you have used a more environmentally friendly mode of transportation?’ when travelling to national conferences (**F**) and international conferences (**G**). Figure 2—source data 1.Numbers of conferences attended and what modes of transport were used, including whether more environmentally friendly modes of transport were available.

Initially, the total number of conferences attended in 2019 was assessed. On average, respondents had attended 3 conferences in 2019, with doctoral researchers/PhD students attending 2.2, postdoctoral researchers attending 3.4, and group leaders/PIs attending 4.8 (Figure 2A). These data reflect the fact that conference attendance increases with career stages, despite networking and getting to know people being of particular importance to early-career researchers.

### Number of conferences attended, modes of transport and sustainability

The survey asked about the number of national and international conferences attended in 2019, the mode of transport used, and the availability of more sustainable travel options. On average, respondents attended more national (2.1) than international (1.4) conferences (Figure 2B,C). 55% travelled by train to national conferences, with 11% going by bus. The main mode of transport to international conferences was air travel (54%), followed by train travel (37%; see Figure 2D,E). Surprisingly, more scientists travelled to national conferences by bicycle (12%) than by car (8%), although this might be explained by the fact that many of the respondents were based in large cities that often host scientific meetings (such as Berlin, Munich and Leipzig).

73% of the respondents stated that they used the most environmentally friendly mode of transport to attend national conferences, while this figure dropped to 54% for international conferences (Figure 2F,G). 16% stated they could have travelled more sustainably to some of the national conferences; while 8% replied they could have done so for all the national conferences they attended. For international conferences, 15% of participants said they could have used more sustainable transport to some of the meetings they attended, while 10% said they could have done so for all of them. However, 21% of participants preferred not to answer this last question, indicating potential discomfort when confronted with their choices regarding sustainable travel to international conferences. These results indicate that it is not necessarily the mind-set of scientists that requires rethinking, but rather that institutional frameworks and conference formats must change to promote sustainability.

### Scientists are willing to attend fewer conferences to protect the environment

**Figure 3.**
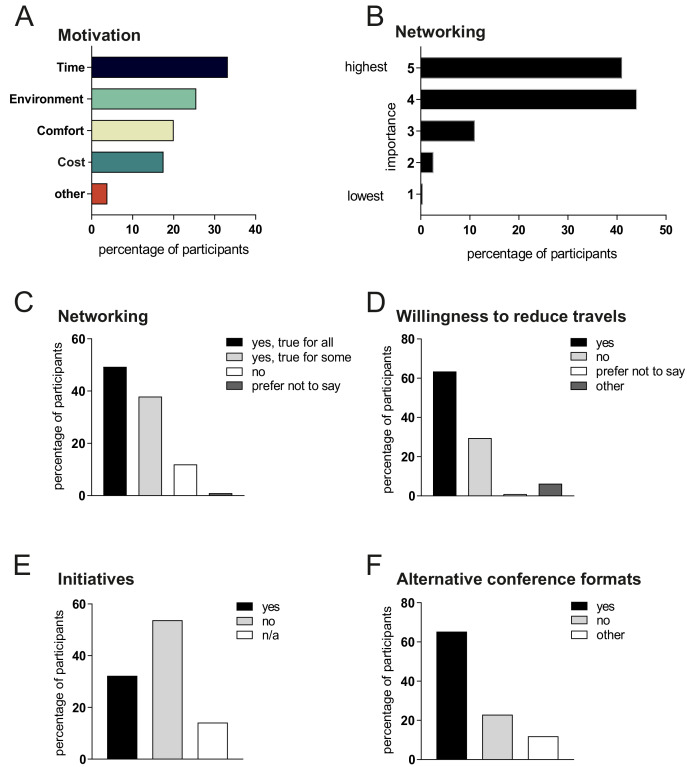
Factors that influence choices in travel to conferences. (**A**) The relative importance of time (black), the environment (green), comfort (chartreuse), cost (cyan) or other (red) when deciding what mode of transport to use to go to a conference. (**B**) Distribution of answers to the question ‘how important do you consider face to face discussions/networking for the scientific community?’ on a scale of 1 (not relevant) to 5 (essential). (**C–F**) Distribution of answers to the following questions: ‘would you say that attending all of the scientific meetings/conferences this year was essential for your career/networking?’ (**C**); "would you be willing to reduce the amount of travelling for your science for the sake of the environment/reducing your personal carbon emission?’ (**D**); ‘are you aware of any initiative of your or any other research institution to promote environmentally friendly business trips?’ (**E**); " Could you imagine alternative web-based concepts for scientific meetings/conferences in the future?’ (**F**). Figure 3—source data 1.Motivations for choosing different forms of travel to conferences.

When asked about the factors that influenced how they decided to travel to conferences, 33% said that time was the most important factor, followed by concerns about the environment (26%), comfort (20%) and cost (18%; see Figure 3A). The survey also asked about the importance of face-to-face discussions/networking in the scientific community on a scale from 1 (not relevant) to 5 (essential); the average score was 4.2, with 85% of respondents answering 4 to 5 (Figure 3B). When asked whether all of the attended conferences in 2019 were essential for their career/networking, 49% agreed, 38% said that some were important, and 12% replied that none had been important for their career/networking (Figure 3C). When asked if they would be willing to reduce their conference travel for environmental reasons, 63% said yes and 30% said no (Figure 3D).

To explore the role of institutions, the survey asked about institutional initiatives to promote environmentally friendly business trips: only 32% of respondents were aware of such initiatives in their own institution, and 54% were not aware of such initiatives, which suggests that institutions need to do more in this area (Figure 3E). A majority of respondents (65%) were also open to the idea of web-based alternatives to traditional conferences, although 23% were not in favour of such alternatives (Figure 3F).

## Advice for 2020 and beyond

It seems unrealistic to expect scientists to stop traveling to conferences and other events altogether. This is especially true in an academic environment that perceives air travel as a driver for academic success despite the lack of evidence supporting this claim (Nursey-Bray et al., 2019; Wynes et al., 2019). This means that, as we explore web-based alternatives to conferences, we must also seek to reduce the carbon footprint of all remaining scientific travel (Favaro, 2014). Actions that employers, institutions and conference organizers could take include the following:

- Promotion of modes of transport with lower carbon emission by, for example, providing train season tickets; by refusing to pay for flights when reasonable alternatives are available; and by committing to an overall reduction in air travel.
- Counting conference travel time as work time (since more sustainable forms of travel can be more time-consuming, e.g. taking the train instead of flying).
- Optimizing conference locations to minimize greenhouse gas emissions (Stroud and Feeley, 2015).
- Raising awareness through, for example, conferences on the topic (such as the 'Reducing Academic Flying' symposium organized by the University of Sheffield in November 2019).
- Acting as role models by globally aiming to reduce carbon emissions through, for instance, transnational agreements between research institutions, starting with voluntarily joined academic partnerships mutually monitoring their own air travel (Caset et al., 2018).
- Carbon offsetting of international flights. Although this policy is being considered, its efficacy is in question, and therefore at this point not further covered (Anderson, 2012).

Policies promoting sustainable travel to scientific conferences have already been implemented by Durham University (UK), Ghent University and KU Leuven (both in Belgium), and other institutions. Durham implemented a sustainable travel plan called the Environmental Sustainability Action Plan 2017–2020 including discounts for sustainable travel options. Ghent University published its Sustainable Travel Policy (Ghent University Website), which includes lists of cities to which travel by plane is either discouraged or not funded. Necessary flights are only compensated upon agreement with CO2logic, an initiative Ghent University collaborates with for deciding which projects will receive financial support. KU Leuven includes support for sustainable travel to conferences and for video conferencing in its Strategic Plan for KU Leuven in 5 Projects. A case study examining the carbon footprint of a complete PhD project reported video conferencing could have reduced the climate change impact of the project by up to 44% (Achten et al., 2013).

### New carbon-conscious conference formats

Rethinking academic travelling in the light of sustainability also requires reframing our current concept of scientific conferences as carbon-conscious. Besides reducing the frequency of conferences (Nathans and Sterling, 2016), concepts for new conference formats include experimenting with virtual platforms. Here, we provide some examples of what new conference formats might look like:

- Virtual conferencing. While it will not be possible to change all existing conferences into virtual events, there are some pioneering examples that we can learn from, such as the Nearly Carbon Neutral (NCN) Conference organized by Ken Hiltner of UC Santa Barbara. He provides a practical guide for running an NCN, based on pre-recorded talks, and therefore being independent of time differences and people’s schedules. Q and A discussions are open several weeks, to allow participants to watch the talks and ask questions in their own time. Another example is neuromatch, an online conference in Computational Neuroscience that was organized in March this year (Goodman et al., 2020).
- Hybrid conferences. It is also possible to combine a virtual interface with regional conference hubs. This concept is based on the idea of a scientific association or society convening at multiple sites, allowing for in-person sessions and workshops. At the same time, digital links between the regional sites would allow all attendants to participate in major events (such as keynote). In November 2019, for example, talks at a meeting organized by the European Biological Rhythms Society were broadcast from Munich to five major hubs and 69 other sites around the world. The Society for Cultural Anthropology took a similar approach when organizing the Displacements conference in 2018, and increased the number of attendees by a factor of six compared with previous years. A second version of this conference, Distribute 2020, will take place in early May.
- Decentralized big conferences. Creating regional conference hubs that are reachable by more sustainable modes of transport is a promising and less radical alternative that still guarantees face-to-face networking. In this setting, intra-institutional, local or national collaborations could be formed.
- Virtual networking formats. Many scientists claim that informal conversations during coffee breaks or receptions are crucial for setting up collaborations or learning about job opportunities at scientific meetings. However, maybe it is time to experiment with new virtual networking concepts that might offer even more of these possibilities. Tele-networking, using video-conference platforms, can take place more frequently than traditional conferences, potentially allowing for better networking opportunities. Similar to coffee breaks or receptions, satellite events gathering special interest groups between or after the conference talks could be organized as virtual social events using Twitter interactive hubs, Slack channels or other virtual platforms. International Slack channels have already been established for certain research areas, such as for the Open Data Science Community (ODSC). These channels enable constant communication and create opportunities for networking and collaboration between scientists.

## Conclusion

In addition to reducing the carbon footprint of scientists, making conferences more virtual could have other advantages. Science could become more inclusive, and thus fairer, because scientists who are not well funded (such as early-career researchers and scientists from countries with limited research funding) and scientists who find it difficult to travel (due to family, personal or health reasons) will get the chance to attend virtual meetings. Additionally, fewer hours spent on planes and at airports will free up time for many other activities (Nathans and Sterling, 2016), both at home and at work. The challenges the scientific community is currently facing due to the COVID-19 pandemic might spawn additional new concepts for building a more sustainable and equitable global scientific community (Weissgerber et al., 2020). If scientists and their institutions and the bodies that organize conferences can get their acts together, the benefits to science and the environment could be far-reaching.

## Methods

**Figure 4.**
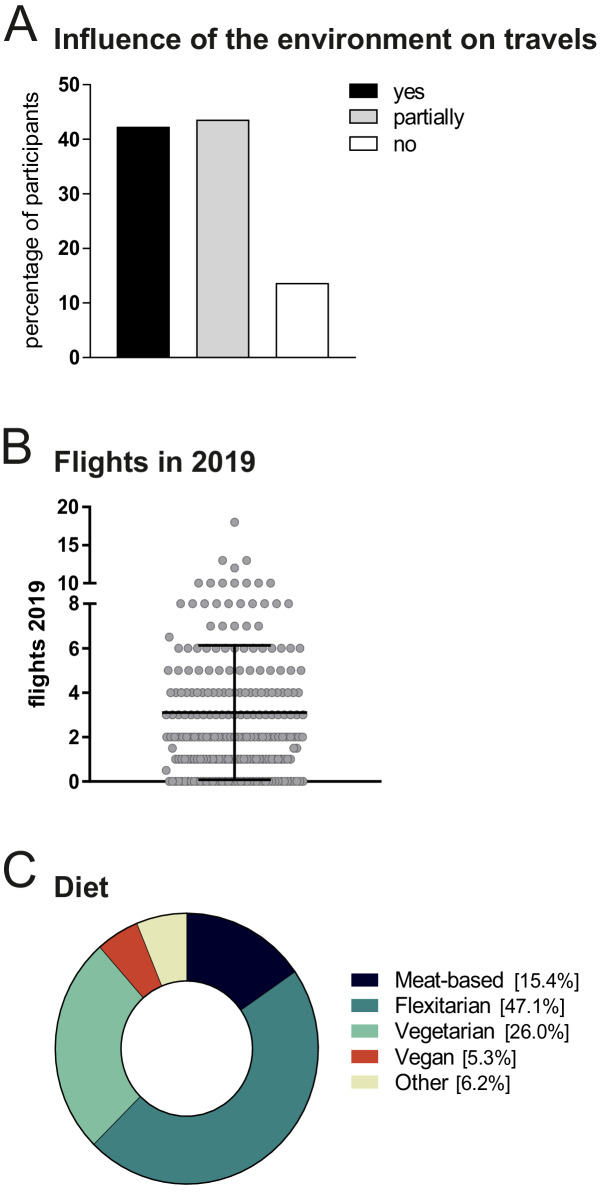
Views on the environment, travel and diet. (**A**) When asked ‘would you say that the environment/climate change affects your travelling behaviour?", 42% of respondents said yes, 44% said partially, and 14% said no. Only one person said they did not care. (**B**) Number of total flights (business and personal) taken by all respondents in 2019. Each dot represents one respondent; mean ± SD. (**C**) Preferred diet of respondents: meat-based (black); flexitarian, (cyan); vegetarian (green); vegan (red); other (chartreuse). Figure 4—source data 1.Opinions on the environment, travel and diet.

The survey (Supplementary file 1) was created using the online tool SurveyPlanet and was conducted using convenience sampling with dissemination via forwarded email invitations or via LinkedIn, and remained open for four weeks. A pilot version of the survey was originally conducted with 8–12 doctoral researchers/PhD students of the Max Delbrück Center for Molecular Medicine in Berlin. Based on this pilot run, some questions were revised. During the analysis of the final survey, seven of the research areas asked about in question 6 (Life Sciences, Neuroscience, Immunology, Microbiology, Genetics, Cardiovascular/Metabolic research and Cancer Biology) were combined into a single Life Sciences research area. 280 respondents completed the survey. Since 227 of these respondents were based in Germany, all the descriptive statistics reported in this article are for these 227 respondents. Researchers in the Life Sciences and doctoral researchers/PhD students are over-represented in the sample, likely due to methods used to disseminate the survey. However, since the next generation of senior scientists will come from the early-career researchers of today, we feel that it is worth reporting their views. Our sample also includes rather high percentages of nutrition-conscious individuals (78% are flexitarian, vegetarian or vegan, and only 15% are meat-eaters; Figure 4C) and environmentally-conscious individuals (although the average number of round-trip flights per respondent was 3.1 in 2019; Figure 4B), which might skew some of the results. Moreover, it is possible to travel from Germany to many other European countries by train, and the same is not true in many countries outside Europe.

## Data Availability

All data generated or analyzed during this study are included in the manuscript and supporting files. The following dataset was generated: HaageV2020A survey of scientists travel behaviour in 2019Dryad Digital Repository10.5061/dryad.m0cfxpp0v

## Additional files

Supplementary file 1. Survey questionnaire. File containing the questions distributed to the survey participants. In questions that were not multiple choice, ‘Essay’ indicates that the participants could write in an answer.
