# Supplementary material for: A survey of travel behaviour among scientists in Germany and the potential for change
Source: eLife. 2020 May 28;9:e56765. doi: 10.7554/eLife.56765 (PMC7255798; doi:10.7554/eLife.56765)
Supplement: Supplementary file 1. — File containing the questions distributed to the survey participants. In questions that were not multiple choice, ‘Essay’ indicates that the participants could write in an answer. [file elife-56765-supp1.docx]

**Question 1:**

Before getting started, would you say that the environment/climate change affects your travelling behaviour?

Choice

- yes
- partially
- no
- I don´t care
- prefer not to say

**Question 2:**

Which gender identity do you identify most with?

Choice

- Female
- Male
- Gender variant/ non-conforming
- not listed
- prefer not to answer

**Question 3:**

Which country are you from?

Essay

**Question 4:**

In which country are you currently living and working?

Essay

**Question 5:**

How many years have you been working in research?

Essay

**Question 6:**

What is your area of research?

Choice

- Life Sciences
- Social Sciences
- Chemistry
- Physics
- Neuroscience
- Immunology
- Microbiology
- Genetics
- Cancer Biology
- Cardiovascular/Metabolic research
- Clinical Research
- Systems Biology

**Question 7:**

What is your academic position?

Choice

- PhD student
- Postdoc
- PI/Group leader

**Question 8:**

How many scientific meetings/conferences have you visited this year (2019)?

Scale 0-20

**Question 9:**

How many **national** scientific meetings/conferences have you visited this year (2019)?

Scale 0-20

**Question 10:**

Which mode of transport did you use for attending national scientific meetings/conferences in 2019?

Choice

- Plane
- Train
- Car (your own)
- Car (rented or shared)
- Bus
- Bike

**Question 11:**

Could you have used a more environmentally friendly mode of transportation for getting to national scientific meetings/conferences?

Choice

- yes, true for all of them
- yes, true for some of them
- no
- prefer not to say

**Question 12:**

How many **international** scientific meetings/conferences did you visit this year (2019)?

Scale 0-20

**Question 13:**

Which mode of transport did you use for attending international scientific meetings/conferences in 2019?

Choice

- Plane
- Train
- Car (your own)
- Car (rented or shared)
- Bus

**Question 14:**

Could you have used a more environmentally friendly mode of transportation for getting to international scientific meetings/conferences?

Choice

- yes, true for all of them
- yes, true for some of them
- no
- prefer not to say

**Question 15:**

Are you aware of any initiative of your or any other research institution to promote environmentally friendly business trips?

Essay

**Question 16:**

What is/was your motivation to choose your mode of transport?

Choice

- Money
- Time
- Comfort
- Environment
- Other

**Question 17:**

Would you say that attending all of the scientific meetings/conferences this year was essential for your career/networking?

Choice

- yes, true for all of them
- yes, true for some of them
- no
- prefer not to say

**Question 18:**

Would you be willing to reduce the amount of travelling for your science for the sake of the environment/reducing your personal carbon emission?

Choice

- yes
- no
- prefer not to say
- other

**Question 19:**

How important do you consider face to face discussions/networking for the scientific community?

Scoring 1-5 (1= not relevant; 5= essential)

**Question 20:**

Could you imagine alternative web-based concepts for scientific meetings/conferences in the future?

Choice

- yes
- no
- other

**Question 21:**

What is your preferred diet?

Choice

- Meat-based
- Flexitarian
- Vegetarian
- Vegan
- Other

**Question 22:**

How many flights (roundtrips) have you taken this year (2019)?

Essay

**Question 23:**

Anything that you would like to add or comment after completing this survey?

Essay
